# Nonlinear Recurrent Neural Network Predictive Control for Energy Distribution of a Fuel Cell Powered Robot

**DOI:** 10.1155/2014/509729

**Published:** 2014-02-20

**Authors:** Qihong Chen, Rong Long, Shuhai Quan, Liyan Zhang

**Affiliations:** ^1^School of Automation, Wuhan University of Technology, Wuhan 430070, China; ^2^College of Science, Huazhong Agricultural University, Wuhan 430070, China

## Abstract

This paper presents a neural network predictive control strategy to optimize power distribution for a fuel cell/ultracapacitor hybrid power system of a robot. We model the nonlinear power system by employing time variant auto-regressive moving average with exogenous (ARMAX), and using recurrent neural network to represent the complicated coefficients of the ARMAX model. Because the dynamic of the system is viewed as operating- state- dependent time varying local linear behavior in this frame, a linear constrained model predictive control algorithm is developed to optimize the power splitting between the fuel cell and ultracapacitor. The proposed algorithm significantly simplifies implementation of the controller and can handle multiple constraints, such as limiting substantial fluctuation of fuel cell current. Experiment and simulation results demonstrate that the control strategy can optimally split power between the fuel cell and ultracapacitor, limit the change rate of the fuel cell current, and so as to extend the lifetime of the fuel cell.

## 1. Introduction

As the rapid development of modern industrial technology, Ocean technology, and space technology, more and more mobile robots are demanded in these areas. Because of the advantages in operating time, weight, and dimensions, proton exchange membrane (PEM) fuel cells have been considered as alternative power sources for mobile robots.

A mobile robot usually has multiple freedoms, which cause the electric load drastically to fluctuate. Sudden changes in power may significantly reduce the operating life of fuel cells in a long term [1, 2]. Furthermore, fuel cells have the characteristics of unidirectional power flow and they cannot absorb the energy from regenerative braking of a robot. As a result, fuel cells are usually arranged with auxiliary power sources to form hybrid power systems and drive mobile robots. Ultracapacitors are highly suitable for the bulk of the transient power demands since the charge/discharge current of an ultracapacitor can vary in a wide range. In this paper we choose a bank of ultracapacitors as auxiliary power source.

A smart power split strategy is indispensable to enhance performance and lifetime of the hybrid power system. Jiang et al. [3] presented an adaptive control algorithm that adjusted the output current set point of the fuel cell. Ferreira et al. [4], Li et al. [5], and Kim et al. [6] developed a fuzzy controller to optimally distribute the power between the fuel cell and the battery. Rodatz et al. [7] designed an optimal control strategy to minimize the hydrogen consumption in a hybrid fuel cell system. Paladini et al. [8] proposed an optimal control strategy to power a vehicle with both fuel cell and battery to reduce fuel consumption. Lin et al. [9] studied a dynamic programming (DP) algorithm based on the fuel consumption and exhaust gas emission for a parallel electric vehicle. These strategies are effective in dealing with system efficiency but address little the lifetime of the fuel cell stack due to rapid load demand variations. Zhang et al. [10] presented a wavelet-transform algorithm to identify and allocate power demands with different frequency contents to corresponding sources to achieve an optimal power management control algorithm. This algorithm can protect fuel cell effectively but is complex and difficult to apply online. Xu et al. [11, 12] and Simmons et al. [13] proposed optimal real-time energy management strategies for a proton electrolyte membrane (PEM) fuel cell bus based on the Pontryagin's Minimal Principle and the determined dynamic programming (DDP). Ziogou et al. [14] deployed a dynamic optimization approach based on nonlinear model of fuel cell. Li et al. [15] developed a constrained model predictive control of a solid oxide fuel cell based on genetic optimization.

Undoubtedly, the fuel cell power systems are nonlinear. Therefore, the global optimization based energy management strategies depend on nonlinear models of the fuel cell power systems and are time costly. Model predictive control (MPC) has been recognized as a powerful methodology for controlling a wide class of nonlinear dynamic system [16]. In this paper we use MPC appropriately, distribute power between the fuel cell and ultracapacitor, avoid frequent fluctuation of fuel cell current, and so enhance the transient performance and extend the operating life of the hybrid system.

There have been three main methods for nonlinear system modeling and predictive control [17]. The first one uses a piecewise linearization to describe the nonlinear behavior of a system. Each model is effective only in a small region, which results in that a mass of models is required [18]. The second one directly employs nonlinear models, but these involve a nonlinear online optimization problem with constraints, which is usually time-consuming and may even be unable to guarantee a feasible solution for real time control [19]. The third method is to use a local linearization approach representing a nonlinear plant, which is valid and simplifies the implement [20–24].

This paper proposes an ARMAX (Autoregressive Moving Average with Exogenous input) modeling approach for fuel cell power systems. Time-variant coefficients of the ARMAX model are estimated by a recurrent neural network. The RNN-ARMAX model is an equal linear model of the fuel cell power system. Therefore, we design linear constrained model predictive control based on the RNN-ARMAX model for the nonlinear fuel cell power system. The design and implementation of the controller are significantly simplified and the method can protect fuel cell from substantial fluctuation of current by trading off transient current demand from the fuel cell to the ultracapacitor, according to constraints and weighting matrices of the output errors.

The remainder of this paper is organized as follows.  Section 2 describes RNN-ARMAX modeling of the fuel cell power system. MPC is designed in Section 3. In Section 4, we implement and discuss simulation results. Conclusions are given in Section 5.

## 2. RNN-ARMAX Modeling

We aim at the optimization of electric power distribution between the fuel cell and ultracapacitor of a fuel cell robot.

### 2.1. System Structure and Description

**Figure 1:**
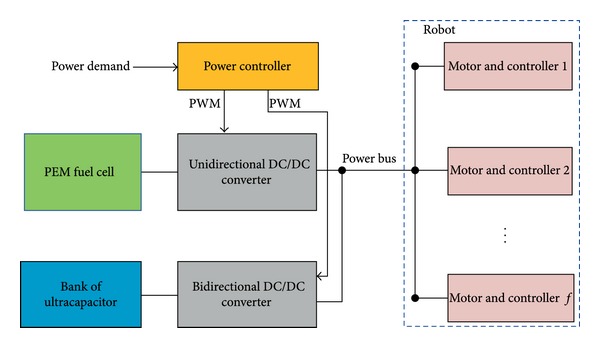
Fuel cell power system of a robot.

The fuel cell power system studied in this paper, as shown in Figure 1, is designed for a mobile robot. The electrical output of the PEM fuel cell is connected to the load through a unidirectional DC/DC converter, and an ultracapacitor bank is also connected to the load through a bidirectional DC/DC converter to form a hybrid fuel cell system. The ultracapacitor bank should supply peak power and be recharged by the fuel cell.

The distribution of power between the fuel cell and the ultracapacitor depends on the duty ratio of the DC/DC converters. Duty ratio of a DC/DC converter is defined as the ratio of switch on time interval, *T*_ON_, to switching period *T*; that is,

(1) $$\begin{array}{c} d=\frac{T_{\text{ON}}}{T}. \end{array}$$

There is one duty ratio, *d*_fc_, in the unidirectional DC/DC converter for controlling output power of the fuel cell. In the bidirectional DC/DC converter, one duty ratio, *d*_*c*_, is for charging the ultracapacitor, and the other, *d*_*d*_, is for discharging the ultracapacitor. Power distribution is optimized by controlling the three duty ratios.

### 2.2. Identification

The hybrid system is a multiple input and multiple output nonlinear system. The control input variables are three duty ratios of the power converters. Input variables are expressed as

(2) $$\begin{array}{c} u\left(t\right)={\left(\begin{array}{ccc} d_{\text{fc}} & d_{d} & d_{c} \end{array}\right)}^{T}. \end{array}$$

The output variables contain output voltage of the fuel cell and the state of charge of the ultracapacitor and so forth. Output variables are chosen as

(3) $$\begin{array}{c} y\left(t\right)={\left(\begin{array}{ccc} V_{\text{fc}} & I_{\text{fc}} & I_{c} \end{array}\begin{array}{ccc} \text{SOC} & V_{b} & I_{b} \end{array}\right)}^{T}, \end{array}$$

where *V*_fc_ is voltage of the fuel cell, *I*_fc_ is current of the fuel cell, *I*_*c*_ is current of the ultracapacitor, SOC is state of charge of the ultracapacitor, *V*_*b*_ is the bus voltage: and *I*_*b*_ is the bus current, respectively. Power demanded by the load, *P*_*d*_, is viewed as a disturbance to the system. We can describe the model as the following nonlinear function:

(4) y(t)=f(φ(t))+ξ(t),

(5) φ(t)=[yT(t−1)…yT(t−n)  uT(t)  …uT(t−m+1)]T≜[φi(t)][n∗ny+(m+1)∗nu]×1,

where  *φ*(*t*)  is the regression vector with known order *n* and *m*,  *n*_*y*_  and  *n*_*u*_  are dimensions of output and input,  *ξ*(*t*)  is the system disturbance, and  *f*(·)  is an unknown nonlinear function, respectively.

If we design MPC based on direct use of the nonlinear model, it involves the online solution of a higher order nonlinear optimization problem with constraints, which is usually computationally expensive and may even be unable to guarantee a feasible solution for real time control.

Here we use RNN-ARMAX to model the system. Performing Taylor expansion on the nonlinear function  *f*(*φ*(*t*))  around the region  *φ*(*t*) = 0  as

(6) y(t)=f(0)+f′(0)φ(t)+12φT(t)f′′(0)φ(t)+… +ξ(t).

We introduce the notation

(7) $$\begin{array}{c} y_{0}=f\left(0\right), \end{array}$$

(8) Θ(φ(t))=(f′(0)+12φT(t)f′′(0)+…)T=[a1,t  Ta2,t  T…  an,t  Tb0,tT  …  bm,t  T]T,

where Θ ∈ **R** ^*n*_*y*_×[*n*∗*n*_*y*_+(*m*+1)∗*n*_*u*_]^ and the coefficients  *a*_*i*,*t*_ = *a*_*i*_(*φ*(*t*)), *b*_*i*,*t*_ = *b*_*i*_(*φ*(*t*))  are nonlinear function of  *φ*(*t*).

We have a regression form of the system described by (4) as follows:

(9) $$\begin{array}{c} y\left(t\right)=y_{0}+\Theta \left(\varphi \left(t\right)\right)\varphi \left(t\right)+\xi \left(t\right). \end{array}$$

**Figure 2:**
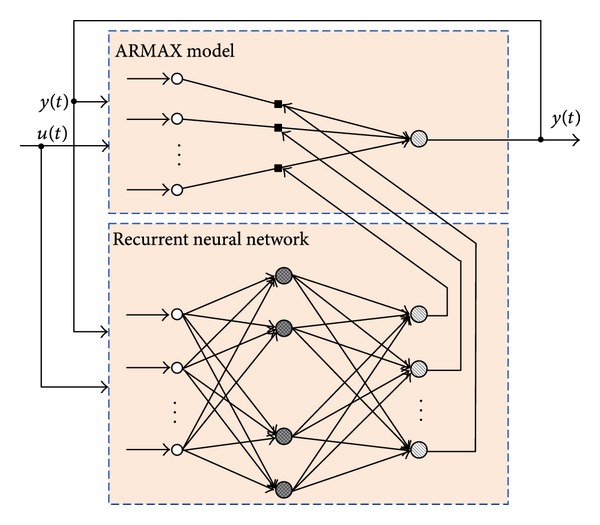
RNN modeling principle.

Here the parameter vector  Θ(*φ*(*t*))  is time variant. The recurrent neural network (RNN) that consists of feed-forward and feedback connections is well known to be capable of modeling and control nonlinear system. We use RNN to estimate  Θ(*φ*(*t*)). The recurrent neural network modeling principle is shown in Figure 2.

The RNN is expressed as

(10) $$\begin{array}{c} O\left(t\right)=KS\left(t\right),\\ S\left(t\right)=\Gamma \left(W\varphi \left(t\right)+\overset{-}{W}S\left(t-1\right)\right),\\ \Gamma \left(x\right)=\left[\sigma \left(x_{1}\right)\;\sigma \left(x_{1}\right)\ldots \;\sigma \left(x_{n_{h}}\right)\right],\\ \sigma \left(x_{j}\right)=\frac{1}{1+e^{-x_{j}}}, \end{array}$$

where *O*(*t*) ∈ **R** ^*n*_*y*_[*n*∗*n*_*y*_+(*m*+1)∗*n*_*u*_]^  is output of the RNN and$\;K,W,\overset{-}{W}\;$are weights for the RNN among the output layer, the input layer, and the hidden layer. Define *n*_*o*_, *n*_*i*_, and *n*_*h*_ as the node amounts of the output layer, the input layer, and the hidden layer, respectively.  *K*, *W*  and $\overset{-}{W}$are expressed as

(11) K=[k1  k2…  knh]no×nh,    S(t)=[si(t)]nh×1,W=[wlk]nh×ni,  W−=[w−lk]nh×nh,no=ny[n∗ny+(m+1)nu], ni=  n∗ny+(m+1)nu.

Then the output of the system is predicted by

(12) $$\begin{array}{c} \hat{y}\left(t\right)=y_{0}+\Psi \left(t\right)O\left(t\right), \end{array}$$

where  Ψ(*t*) ∈ **R** ^*n*_*y*_×*n*_*y*_[*n*∗*n*_*y*_+(*m*+1)∗*n*_*u*_]^ and

(13) $$\begin{array}{c} \Psi \left(t\right)=\left[\begin{array}{cccc} \varphi^{T}\left(t\right) & 0 & 0 & 0\\ 0 & \varphi^{T}\left(t\right) & 0 & 0\\ \vdots & \vdots & \vdots & \vdots \\ 0 & 0 & \cdots & \varphi^{T}\left(t\right) \end{array}\right]≜\left[\begin{array}{c} \Psi_{1}\left(t\right)\\ \Psi_{2}\left(t\right)\\ \vdots \\ \Psi_{n_{y}}\left(t\right) \end{array}\right]. \end{array}$$

The performance criterion  *ψ*(*t*)  of the neural network is then defined by

(14) $$\begin{array}{c} \psi \left(t\right)=\frac{1}{2}{\left(y\left(t\right)-y_{0}-\Psi \left(t\right)O\left(t\right)\right)}^{T}\left(y\left(t\right)-y_{0}-\Psi \left(t\right)O\left(t\right)\right), \end{array}$$

where *y*(*t*) is sampled output of the system. Therefore, the weights are adjusted to reduce the cost function  *ψ*(*t*)  to a minimum value by the gradient descent method. The weight vectors are updated along with

(15) K(t+1)=K(t)−η∂ψ(t)∂K,W(t+1)=W(t)−η∂ψ(t)∂W,W−(t+1)=W−(t)−η∂ψ(t)∂W−,

where  *η*  is a positive learning rate.

Let *q*, *r* be the quotient and remainder of *i*/[*n*∗*n*_*y*_ + (*m* + 1)*n*_*u*_], respectively. If *h* = 0, then set *r* = [*n*∗*n*_*y*_ + (*m* + 1)*n*_*u*_]. Else set *q* = *q* + 1.  ∂*ψ*(*t*)/∂*K*, ∂*ψ*(*t*)/∂*W*, and  $\partial \psi (t)/\partial \overset{-}{W}$  are then calculated as follows:

(16) ∂ψ(t)∂kij=(y0,q+Ψq(t)O(t)−yq(t))sj(t)φr(t),∂ψ(t)∂wij=(y0+Ψ(t)O(t)−y(t))Ψ(t)ki(t)Hi(t)φj(t),∂ψ(t)∂w−ij=(y0+Ψ(t)O(t)−y(t))Ψ(t)ki(t)Hi(t)sj(t−1),

where

(17) $$\begin{array}{c} H_{i}\left(t\right)=\frac{e^{-h_{i}(t)}}{{\left(1+e^{-h_{i}(t)}\right)}^{2}},\\ h_{i}\left(t\right)=\overset{n_{i}}{\underset{l=1}{\sum }}w_{il}{\left(t\right)\varphi }_{l}\left(t\right)+\overset{n_{h}}{\underset{l=1}{\sum }}{\overset{-}{w}}_{il}\left(t\right)s_{l}\left(t-1\right). \end{array}$$

The update rules of (15) call for a proper choice of the learning rate  *η*. For a small value of  *η*  the convergence is guaranteed but the speed is slow; if  *η*  is too big, the algorithm becomes unstable. Here we develop a guideline in selecting the learning rate properly. A discrete Lyapunov function is given by

(18) $$\begin{array}{c} V\left(t\right)=\frac{1}{2}e^{T}\left(t\right)e\left(t\right), \end{array}$$

where

(19) $$\begin{array}{c} e\left(t\right)=y\left(t\right)-y_{0}-\Psi \left(t\right)O\left(t\right). \end{array}$$

Thus the change of Lyapunov function due to the training process is obtained by

(20) ΔV(t)=V(t+1)−V(t)=12[eT(t+1)e(t+1)−eT(t)e(t)].

The error difference due to the learning is represented by

(21) $$\begin{array}{c} e\left(t+1\right)=e\left(t\right)+\Delta e\left(t\right)=e\left(t\right)+{\left[\frac{\partial e(t)}{\partial W}\right]}^{T}\Delta W, \end{array}$$

where  Δ*W*  represents a change in an arbitrary weight vector.

From the update rule (15),

(22) $$\begin{array}{c} \Delta W=-\eta \frac{\partial \psi \left(t\right)}{\partial W}=\eta \frac{\partial O^{T}\left(t\right)}{\partial W}\Psi^{T}\left(t\right)e\left(t\right). \end{array}$$

Then we have the following general convergence theorem.

**Theorem 1**

*η*  is the learning rate for the weights of RNN and  ||·||  is the usual Euclidean norm in  *R* ^*n*^. Then the convergence is guaranteed if  *η*  is chosen as

(23) $$\begin{array}{c} 0<\eta <\frac{2}{\max_{t}{||\Psi \left(t\right)\left(\partial O(t)/\partial W\right)||}^{2}}. \end{array}$$

**Proof**

From equations (20)–(22),  Δ*V*(*t*)  can be calculated as

(24) ΔV(t)=ΔeT(t)[e(t)+12Δe(t)]=[[∂e(t)∂W]Tη∂OT(t)∂WΨT(t)e(t)]T ×{e(t)+12[∂e(t)∂W]Tη∂OT(t)∂WΨT(t)e(t)}=−eT(t){ηΨ(t)∂O(t)∂W∂OT(t)∂WΨT(t)     −12Ψ(t)∂O(t)∂W∂OT(t)∂WΨT(t)     ∗Ψ(t)∂O(t)∂W∂OT(t)∂WΨT(t)}e(t)=−12η[∂OT(t)∂WΨT(t)e(t)]T ×{2I−η∂OT(t)∂WΨT(t)Ψ(t)∂O(t)∂W} ×[∂OT(t)∂WΨT(t)e(t)].

To guarantee  Δ*V*(*t*) < 0,  *η*  should satisfy the following inequality

(25) $$\begin{array}{c} 2I-\eta \frac{\partial O^{T}\left(t\right)}{\partial W}\Psi^{T}\left(t\right)\Psi \left(t\right)\frac{\partial O\left(t\right)}{\partial W}>0, \end{array}$$

(26) $$\begin{array}{c} \eta >0. \end{array}$$

From inequalities (25) and (26), we obtain

(27) $$\begin{array}{c} 0<\eta \;\underset{t}{\max}{||\Psi \left(t\right)\frac{\partial O\left(t\right)}{\partial W}||}^{2}<2. \end{array}$$

Namely,  *η*  satisfies

(28) $$\begin{array}{c} 0<\eta <\frac{2}{\max_{t}{||\Psi \left(t\right)\left(\partial O(t)/\partial W\right)||}^{2}}. \end{array}$$

This proves the theorem.

We can establish a state space model from the matrix polynomials (7), (8), and (9) by defining a state vector given by

(29) x(t)=[x1,tT  x2,tT…  xn,tT]T,x1,t  =y(t),xk,t=∑i=1n+1−ka^i+k−1,t−1y(t−i)+∑i=1n+1−kb^i+k−1,t−1u(t−i),k  =  2,3,…,n.

A state space model can then be given by

(30) x(t+1)=Atx(t)+Btu(t)+Ξ(t+1),y(t)=Cx(t),

where

(31) At=[a^1,t10⋯0a^2,t01⋯0⋮⋮⋮⋱⋮a^n−1,t00⋯1a^n,t00⋯0],  Bt=[b1,tb2,t⋮bn,t],Ξ(t+1)=[y0+ξ(t+1)0⋮0],  C=[10⋮0]T.

Model (30) is a state space representation of MIMO RNN-ARX model (4). The parameters in  *A*_*t*_ and  *B*_*t*_  are estimated by the RNN, and the state  *x*(*t*) at time *t* can be easily obtained by (29) according to the present output *y*(*t*), the past input/output data, and output of the RNN.

## 3. Controller Design 

A predictive controller will be designed to predict the output trajectory of the fuel cell power system and compute a series of control actions, subject to constraints, that will minimize the difference between the predicted trajectory and desired trajectory. A prominent advantage of this controller over other control schemes is its ability to deal with constraints in a systematic and straightforward manner.

To design predictive controller for the system, an objective function is defined as [18]

(32) min⁡u(t),⋯,u(t+N−1) ⁡J=∑k=1Nu((y^(t+k)−yr(t+k))T×Q(y^(t+k)−yr(t+k))+uT(t+k)RuT(t+k)),

where *N*_*u*_ is predictive horizon, $\hat{y}\left(t+k\right)$ is the estimated output of the system at instant *t* + *k* through models based on information available at instant*t*. *y*_*r*_(*t* + *k*) is the desired output at instant *t* + *k*, and *Q*, *R* are weighting matrices on output errors and control, respectively. We choose the control horizon to be equal to the prediction horizon and define  *Q* = diag⁡(*Q*_*V*_fc__ *Q*_*I*_fc__ *Q*_*I*_*c*__ *Q*_SOC_ *Q*_*V*_*b*__)  and  *R* = diag⁡(*R*_*d*_fc__ *R*_*d*_*d*__ *R*_*d*_*c*__), where,  *Q*_*V*_fc__, *Q*_*I*_fc__, *Q*_*I*_*c*__, *Q*_SOC_, and *Q*_*V*_*b*__ are penalties on errors in  *V*_fc_, *I*_fc_, *I*_*c*_, SOC and  *V*_*b*_, respectively.  *R*_*d*_fc__, *R*_*d*_*d*__, and  *R*_*d*_*c*__  are penalties on  *d*_fc_, *d*_*d*_ and *d*_*c*_, respectively.

Substituting state equations (30) into (32), the equation is abbreviated as

(33) $$\begin{array}{c} \underset{U}{\min}\;J=\frac{1}{2}{||U||}_{\Omega }^{2}+\left(y^{T}\left(t\right)L+G\right)U, \end{array}$$

where *y*(*t*) is system output at instant *t*, and *Ω*, *L*, *G* are constant matrices calculated through the system model and matrices *Q*, *R*.

Consider the following:

(34) $$\begin{array}{c} U=\left(u^{T}\left(t\right),u^{T}\left(t+1\right),\ldots ,u^{T}\left(t+N_{u}-1\right)\right). \end{array}$$

In the hybrid system, there are several limits to deal with. Rapid variation on current will reduce lifetime of fuel cell, so it is required to constrain the fluctuation of fuel cell current; that is,

(35) $$\begin{array}{c} -\Delta I_{\max}\leq I_{\text{fc}}\left(t+1\right)-I_{\text{fc}}\left(t\right)\leq \Delta I_{\max}, \end{array}$$

where Δ*I*_max⁡_ is the acceptable maximum value. Moreover, the state of charge of the ultracapacitor, the current of the ultracapacitor, and the voltage of the fuel cell should be limited to some expected range:

(36) $$\begin{array}{c} \text{SO}{\text{C}}_{\min}\leq \text{SOC}\leq \text{SO}{\text{C}}_{\max}, \end{array}$$

(37) $$\begin{array}{c} -I_{c,\max}\leq I_{c}\leq {I_{c,}}_{\max}, \end{array}$$

(38) $$\begin{array}{c} V_{\text{fc},\min}\leq V_{\text{fc}}\leq V_{\text{fc},\max}, \end{array}$$

where SOC_min⁡_ and *V*_fc,min⁡_ are the lower limitations, SOC_max⁡_, *I*_*c*,max⁡_, and *V*_fc,max⁡_ are the upper limits, respectively. These limitations are determined by the characteristics of the ultracapacitor and fuel cell.

A prominent advantage of MPC is its ability to deal with constraints. Deduced from equations (30), (32) and inequalities (35)–(38), the control optimization is transformed to the following constrained quadratic programming problem:

(39) min⁡ UJ=12||U||Ω2+(yT(t)L+G)U,s.t.    Umin⁡≤EU≤Umax⁡,

where  *U*_min⁡_, *U*_max⁡_ ∈ *R* ^*m*^, and *E* ∈ *R* ^*m*×*N*_*u*_^ are constant matrices obtained from (30) and inequalities (35)–(38). We can solve this optimal problem using the neural network method investigated in [25].

## 4. Experiment and Simulation

The hybrid fuel cell system, as shown in Figure 1, is designed to power a robot. The rated power is 500 W. The DC bus voltage is controlled around 24 V. The PEM fuel cells have 40 cells and an active area of 22 cm^2^. The ultracapacitor is 200 F and the rated voltage is 24 V. The value of capacitance can be realized by a bank of 8 ultracapacitors, each with capacitance of 1600 F and a rated voltage of 3 V, connected in series. The upper and lower limits of SOC are 1 and 0.45, respectively. The maximum stored energy is 16 W h, although only 12.76 W h is available between the maximum and minimum of SOC. This 12.76 W h corresponds to an average power at 500 W for 92 seconds and that is sufficient to buffer the fuel cell from acceleration transients.

### 4.1. Modeling Experiment and Simulation

When real input and output data of the PEM fuel cell was sampled, the operating parameters are shown in Table 1.

**Figure 3:**
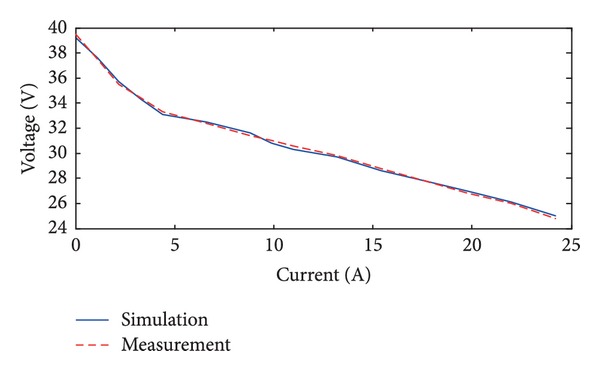
The simulated and measured V-I characteristics curves of the fuel cell.

**Figure 4:**
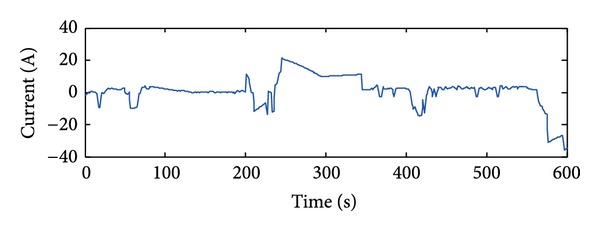
Current of the ultracapacitor.

**Figure 5:**
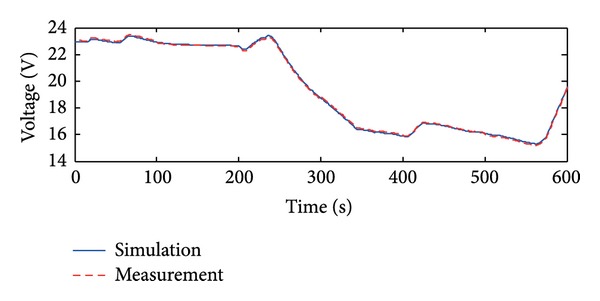
The simulated and measured voltage of the ultracapacitor.

The collected data are equally divided into two groups. The first group is used for modeling and the second group is used for validating. The simulated and measured V-I characteristics curves of the fuel cell are shown in Figure 3. Current of the ultracapacitor changes as Figure 4, and the simulated and measured voltage curves are shown in Figure 5. It is shown that the RNN-ARMAX model closely matches the practical fuel cell power system.

### 4.2. Control Simulation

Control performances of constrained and unconstrained MPCs are studied and compared to validate the proposed constrained MPC. The constraints of the constrained MPC are listed in Table 2.

**Figure 6:**
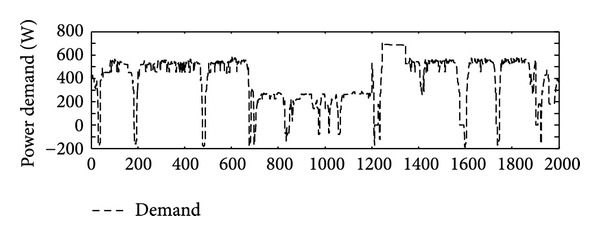
Power profile.

A typical load cycle that is used in simulation and the power profile, as shown in Figure 6, is considered as the power demand.

**Figure 7:**
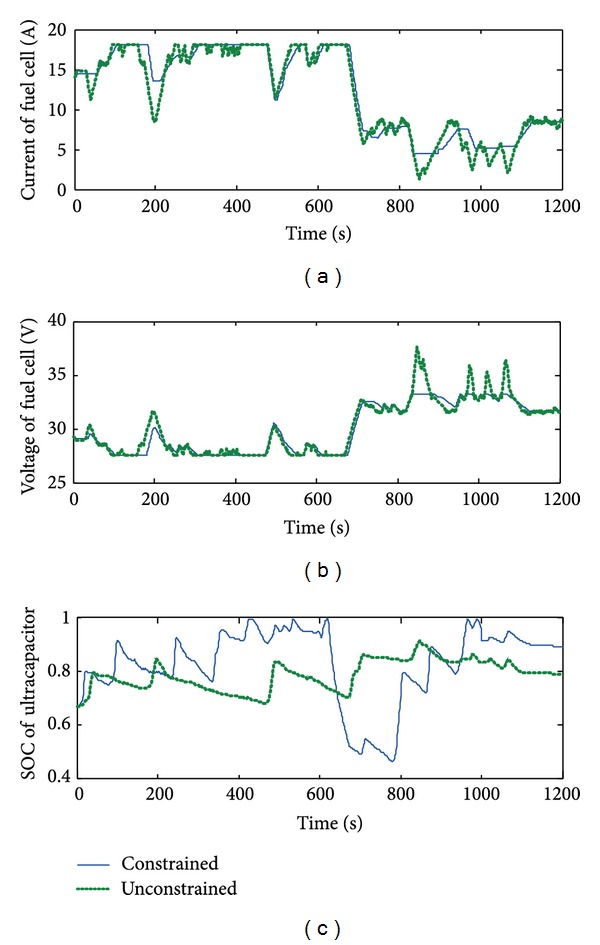
Simulation results of constrained and unconstrained MPC: (a) current of fuel cell; (b) voltage of fuel cell; (c) SOC of ultracapacitor.

The simulation results for both the unconstrained and the constrained MPC are shown in Figure 7. It is shown that, there exist significant perturbations in current of fuel cell for unconstrained MPC. This phenomenon may cause oxygen starvation because the dynamic response of oxygen supply is slower, while in the case of the constrained MPC, current and voltage are much smoother.

In the case of constrained MPC, the oscillation of SOC of the ultracapacitor is much larger than that of the unconstrained MPC. The reason is that constrained MPC draws much more energy from the ultracapacitor to supply the peak load and so limits perturbations of the current of the fuel cell.

**Figure 8:**
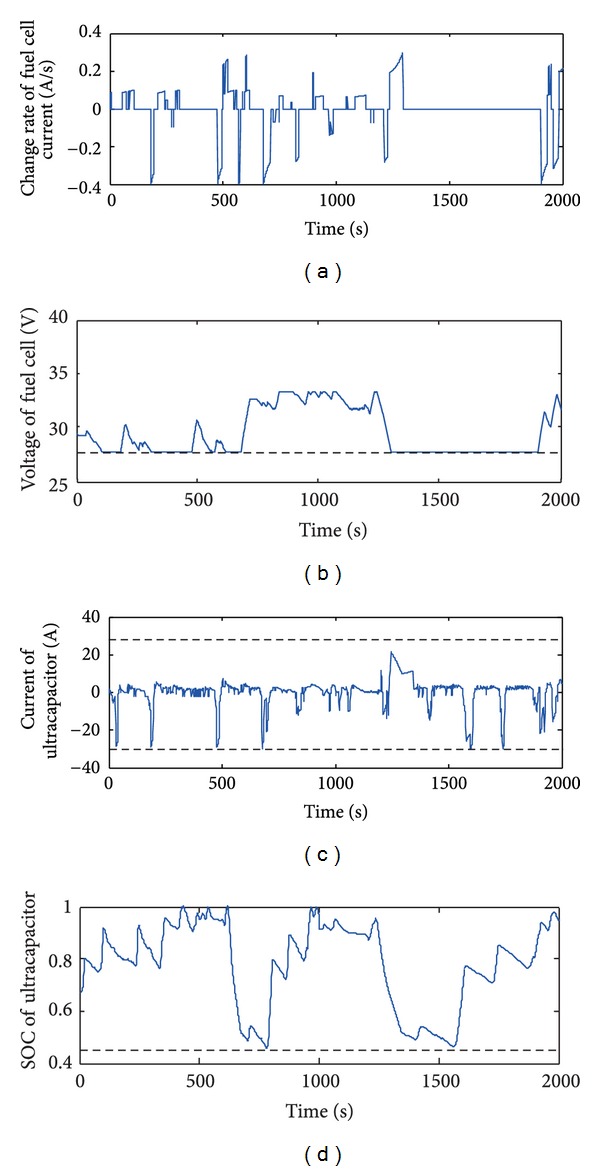
Curves for validating of constraints: (a) change rate of fuel cell current; (b) voltage of fuel cell; (c) current of ultracapacitor; (d) SOC of ultracapacitor.

Constraint results are shown in Figure 8. It's exciting that the maximum rate of change of the fuel cell is 0.4 A/s, the minimum voltage of the fuel cell is 27.5 V, the charge and discharge current of the ultracapacitor are no more than 30 A, and the SOC of the ultracapacitor is between 0.45 and 1. It is shown that these variables change in the desired and constrained ranges. These phenomena demonstrate that the constraints on the fuel cell power system are valid.

**Figure 9:**
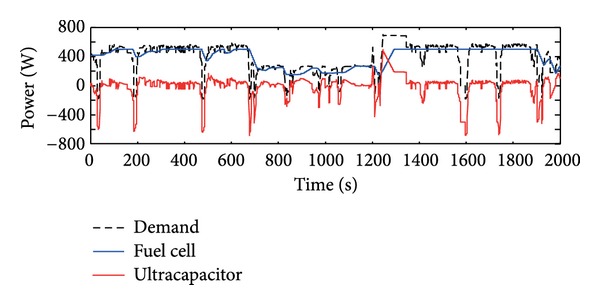
Power distribution of the hybrid system.

The power split under the constrained MPC is shown in Figure 9. We set the minimum voltage of the fuel cell as 27.5 V and the corresponding maximum power of fuel cell as 500 W. It is noticed that the fuel cell power changes in low speed and is no more than 500 W. The high frequency power demands are squeezed from the ultracapacitor. Furthermore, SOC,  *I*_*c*_ and other constrained variables satisfy their constraints. Consequently, the output power of the fuel cell is well controlled and it is helpful to extend the operating life of the fuel cell.

## 5. Conclusions

RNN-ARMAX model was established and linear constrained MPC was developed and verified for a fuel cell power system. The proposed approach, different from other approaches, models the nonlinear fuel cell power system as linear time varying system. Accordingly, linear constrained MPC can be used to globally optimize power distribution and deal with limitations. The design and implementation of the controller are significantly simplified and the method can protect fuel cell from substantial fluctuation of current by trading off transient current demand from the fuel cell to the ultracapacitor.

## Figures and Tables

**Table 1:** Parameters used in the experiment and simulation.

Sym.	Meaning	Value
*T*_st_	Temperature of fuel cell	343 K
*T*_atm_	Atmospheric temperature	295 K
*P*_H_2__	Partial pressure of hydrogen	1.5 atm
*n*	Number of cells in each stack	40
*A*	Active area of fuel cell	22 cm^2^
*C*	Capacitance of ultracapacitor	200 F
*V*_*c*,max⁡_	Rated voltage of ultracapacitor	24 V

**Table 2:** Constraints for the constrained MPC.

Sym.	Meaning	Lower limit	Upper limit
Δ*I*_max⁡_	Rate of change of fuel cell current	−0.4 A/s	0.4 A/s
SOC	State of charge of the ultracapacitor	0.45	1
*I*_*c*_	Current of the ultracapacitor	−30 A	30 A
*V*_st_	Voltage of the fuel cell	27.5 V	40 V
